# Hygiene practices in abattoir and slaughter slab, determinants and assessment of abattoir and slaughter slab facilities in Abakaliki, Ebonyi State South-East Nigeria

**DOI:** 10.4314/ahs.v21i4.50

**Published:** 2021-12

**Authors:** Adaoha Pearl Agu, Cosmas Kenan Onah, Chukwuma David Umeokonkwo, Richard Chukwuka Nnabu, Alfred Friday Igwe Una

**Affiliations:** 1 Department of Community Medicine, Ebonyi State University Abakaliki; 2 Department of Community Medicine, Alex Ekwueme Federal University Teaching Hospital Abakaliki (AE-FUTHA), Ebonyi State; 3 African Institute for Health Policy and Health Systems, Ebonyi State University Abakaliki; 4 Government House Clinic, Government House Abakaliki Ebonyi State

**Keywords:** Knowledge, Hygiene Practices, Abattoir, Slaughter slab, Determinants, Ebonyi, Nigeria

## Abstract

**Background:**

Workers in slaughterhouses engaging in unhygienic practices create conducive environments for zoonoses and meat contamination. Knowledge of hygiene practices and their determinants provides evidence for the design of targeted interventions.

**Objectives:**

We investigated knowledge and determinants of hygiene practices among workers in slaughterhouses and assessed slaughterhouse facilities in Abakaliki.

**Methods:**

Workers in the Central Meat Market abattoir and Slaughter slab Abakaliki were interviewed in a cross-sectional quantitative study to ascertain their knowledge and hygiene practices while abattoir facilities were assessed using a checklist. Associations were analysed with Chi-square while predictors were determined using binary logistic model.

**Results:**

We interviewed 188 workers 75.5% and 85.6% of whom had good knowledge and good hygiene practices respectively. However, hand-washing before and after handling meat (44.1%), cleaning work surfaces with soap and water (45.2%) and sanitary disposal of waste (6.9%) were suboptimal. Knowledge of good hygiene practice was a predictor of good hygiene practice (AOR: 4.6, 95% CI: 2.0–11.3, p=0.001). Well water and borehole were present in both slaughterhouses and cold rooms were available in Central Meat market abattoir.

**Conclusions:**

The level of good knowledge was high and this was a determinant of good hygienic practices. Training on hygiene practices is recommended to prevent meat contamination and zoonoses.

## Introduction

An issue of great interest and growing concern is the spread of infectious diseases that emerge or re-emerge from the interfaces between animals and humans and the ecosystems in which they live. Emerging and existing infectious diseases at the animal-human-ecosystem interface have been of growing concern because of their epidemic and endemic potential as well as their adverse socioeconomic consequences^1^. Key examples are zoonoses and foodborne diseases of animal origin which are of public health and animal health importance. Prevention of these diseases by controlling hazards in meat production processes and improving food safety has been recognized as an effective strategy^2^,^3^.

Hazard analysis control points (HACCP) systems which prevent and reduce food safety hazards through critical control points (CCP)^5^, as well as good hygiene practices, are both parts of an effective food safety management system^6^. Despite the importance of optimum levels of food safety (meat safety) in abattoirs, reports have shown it to be poor in some abattoirs in Nigeria, with interventions needed in the food safety plan^6^,^7^.

Workers in abattoirs who engage in unhygienic practices, create a conducive environment for zoonoses among the workers and contamination of the meat for sale^8^. Unfortunately, the microbial profile of meat in abattoirs and butchery shops in sub-saharan countries including Nigeria, is higher than standards set by World Health Organization (WHO)^9^,^10^ and there have been occurrences of zoonoses among abattoir workers and in cattle in abattoirs across Nigeria^11^–^13^. Studies in Nigeria have also reported substandard facilities, unsanitary environments and poor hygienic practices in abattoirs and slaughterhouses^14^–^20^ even though abattoir sanitation is an essential component of The National Environmental Sanitation Policy of Nigeria^21^.

The focus of the few published research on abattoirs in Ebonyi State has been on isolation of micro-organisms in the environment^22^, antimicrobial resistance to antibiotics^23^,^24^ and isolation of helminths in ruminants^25^. A study^23^ on the assessment of bacteria effluent qualities reported the presence of antibiotic- resistant bacteria in untreated abattoir wastewater at the abattoir in the Central Meat Market, Abakaliki but an assessment of the facilities was outside its scope. A similar study^24^ on the antibiotic susceptibility pattern of Salmonella and Pseudomonas species isolated from the effluents from the Central Meat Market abattoir and the slaughter slab in Abakaliki described the bacterial profile and multidrug-resistant traits of the species found. In contrast, there is much less information on hygienic practices of the workers in these slaughterhouses in Abakaliki.

The purpose of this study was to investigate the knowledge and determinants of hygiene practices among workers in abattoir and slaughter slab in Abakaliki, Ebonyi State and assess the facilities. The findings could serve as a baseline in the design of interventions to protect the meat from contamination and the workers from zoonoses.

## Methods

### Study Area and Design

We conducted a total population cross-sectional quantitative study among workers in the abattoir and slaughter slab in Abakaliki city which spans parts of Abakaliki and Ebonyi Local Government Areas (LGAs) of Ebonyi State, South East Nigeria. Ebonyi State is one of the 36 states of the Federal Republic of Nigeria with its capital as Abakaliki. Abakaliki had a projected population of 172,176 in 2011^26^. The inhabitants are mostly farmers, traders and civil servants. The temperature in Abakaliki varies from 65○F to 89○F^27^. The sliding 31-day rainfall is at least 0.5 inches in the rainy season (February to November) and most rain falls during the 31 days centered around September 22 with an average total accumulation of 8.9 inches^27^.

The study sites were the major meat processing points in Abakaliki city located in the abattoir in the Central Meat Market, Abakpa market (in Ebonyi LGA) and the Slaughter slab on Ogoja road (in Abakaliki LGA). The animals slaughtered in Central Meat Market abattoir are cow, sheep and occasionally goats (180 to 450 animals monthly) while only cows (180 to 360 monthly) are slaughtered at the Slaughter slab. The survey was on the abattoir workers' knowledge and practice of good hygiene and sanitation. All the workers in the selected abattoirs who gave informed consent were eligible for study which took place during their monthly meetings in February and March 2016. Workers who were absent during the February meeting were reached in their workplace in March. People who sell food and other items in and around the premises were excluded from the study.

### Data Collection and Management

Data were collected using pre-tested interviewer-administered questionnaires. The questionnaire was designed by the researchers using published related research^28^,^29^ and reviewed by experts in public health, microbiology and sociology disciplines for content validity. A pre-test of the questionnaire was carried out on ten workers in a nearby town – Ezzamgbo in Ebonyi State and a few of the questions were modified to improve understanding. A double translation of the questionnaire was done between English and the local dialects of the Igbo language. Five trained research assistants with tertiary education in medical and para-medical sciences administered the questionnaire in the local dialect of the Igbo language. The questionnaire had sections on socio-demographic and work characteristics, knowledge, attitude and practices of good hygiene and sanitation. We assessed the availability of the abattoir and slaughter slab facilities using a checklist adapted from the Policy guidelines on market and abattoir sanitation developed by the Nigerian Federal Ministry of Environment^21^.

### Statistical Analysis

Data analysis was carried out with IBM SPSS Statistics version 20. Knowledge questions were scored zero for incorrect and one for accurate response; total overall possible knowledge score was 23. Scores of 11.5 marks and above were graded as good knowledge while those below were graded as poor. Similar to knowledge, practice questions were also scored zero for incorrect and one for an accurate response; scores of 8.5 marks up to a maximum of possible 17 were graded as good practice while those below 8.5 were categorized as poor. The modified Bloom's cut-off was used to categorize these variables^30^. Statistically significant relationships of independent variables with knowledge and practice were determined at p < 0.05 and a cut-off of p=0.2 was the criteria for inclusion of independent variables into binary logistic model for determination of predictors of knowledge and practice.

## Results

All the workers (188) in the abattoir and slaughter slab were interviewed and all responded adequately to the questions, giving a response rate of 100%. One hundred and eighteen (62.8%) of the respondents were -workers in the Central Meat Market abattoir while 70 (37.2%) were in Slaughter slab. The majority of the abattoir workers were males 136 (72.3%) and Christians 168 (89.4%) between the ages of 21 and 40 years 137 (72.9%). The abattoir workers were mostly retailers 93 (49.5%) and butchers 83 (44.1%) and many of them had received training on abattoir work 149 (79.3%) which was majorly provided by their employers 121 (81.2%).

Table 1 shows that majority 161 (85.6%) of the abattoir workers used personal protective equipment (PPE) and apron is the most commonly used. Over 70% of them used these PPE regularly of which 78% of them are provided by the workers themselves. Over 70% of the workers knew proper waste disposal, proper storage of leftover meat and regular hand-washing as food safety and environment safety measures while the least known measure was wiping of surface with soap and water, known only to 43.6%. About 70% agreed that government and health agencies are actively involved in making sure that proper hygiene is observed in the abattoir. Generally, the abattoir workers had good knowledge level of good hygiene practices (75.5%).

**Table 1:** PPE^ usage, reported availability of abattoir facilities and knowledge of good hygiene practices

Variables	Frequency (%)
**Used any PPE**	161 (85.6)
**Frequency of use of PPE**	
Always	119 (73.9)
Sometimes	41 (25.5)
Rarely	1 (0.6)
**Provider of PPE used**	
Self	126 (78.3)
Employer	32 (19.9)
Environmental health officer	3 (1.9)
**Type of PPE used**	
Apron	154 (81.9)
Boot	13 (6.9)
Hand glove	13 (6.9)
Face mask	4 (2.1)
Goggles	2 (1.1)
Cap	2 (1.1)
**Method of abattoir waste disposal known**	
Open dumping	143 (76.1)
Burning	13 (6.9)
Land filling	21 (11.2)
Do not know	11 (5.9)
**Food and environmental safety measures known** *	
Proper waste disposal	151 (80.3)
Proper storage of leftover meat	135 (71.8)
Regular hand-washing	134 (71.3)
Wearing PPE	131 (69.7)
Washing hand after going to toilet	113 (60.1)
Wiping work surface with soap & water	82 (43.6)
**Benefits of working in safe environment known** *	
Enhanced good health	155 (82.4)
Avoidance of disease transmission	130 (69.1)
Avoidance of injuries	104 (55.3)
Enhanced efficiency	63 (33.5)
**Insufficiency of cleaning materials**	
Brooms	37 (61.7
Forks/shovel	12 (20.0)
Soap	11 (18.3)
**Source of water in abattoir**	
Borehole	109 (58.0)
Well	74 (39.4)
Tap	5 (2.7)
**Government and health agencies are actively being involved in** **ensuring good hygiene practice in abattoir**	
Yes	132 (70.2)
No	56 (29.8)
**Knowledge of good hygiene practices and PPE**	
Good	142 (75.5)
Poor	46 (24.5)

* Multiple responses were allowed

^ Personal Protective Equipment

Table 2 shows that open dumping is the commonest waste disposal method reported by the workers (83.5%), while burning, land filling and burying were reported by 9.0%, 5.3% and 1.6% respectively. Majority, 153 (81.4%), of the abattoir workers clean their work surfaces daily and less than half (45.2%) of the respondents used soap and water in the cleaning process. Half of them store meat in cold rooms and a little over 20% use freezers and refrigerators for that purpose. Eight (4.3%) do not use any storage system while 6 (3.2%) leave the meat at room temperature. Similarly, 99 (52.7%) store left-over meat in deep freezers and 79 (42.0%) use refrigerators for storage. Eleven (5.9%) and 10 (5.3%) dry or smoke such left overs respectively. The commonest food safety measures practiced by the abattoir workers while at work include disposal of spoilt meat 183 (97.3%) and avoiding work if they had diarrhea 170 (90.4%). Over 80% avoid work if they had boil or suffered flu respectively. Hand hygiene after using the bathroom was practiced by 135 (71.8%). Washing hands before and after handling meat was not a common practice among them as only 83 (44.1%) observe such practice. However, overall good composite practice was seen in majority (85.6%) of the respondents.

**Table 2:** Reported hygiene practices of abattoir workers

Variable	Frequency (%)
**Method of abattoir waste disposal:**	
Open dumping	157 (83.5)
Burning	17 (9.0)
Land filling	10 (5.3)
Burying	3 (1.6)
Others	1 (0.5)
**Frequency of cleaning work surface**	
Daily	153 (81.4)
After every sale	25 (13.3)
Weekly	8 (4.3)
Monthly	1 (0.5)
Occasionally	1 (0.5)
**Material used to clean work surface:**	
Water only	103 (54.8)
Soap and water	85 (45.2)
**Frequency of cleaning abattoir lairage**	
Daily	137 (72.9)
Weekly	9 (4.8)
Monthly	3 (1.6)
Don't know	37(20.7)
**Where meat is stored**	
Cold room	94 (50.0)
Freezer	41 (21.8)
Refrigerator	39 (20.7)
None	8 (4.3)
Room temperature	6 (3.2)
**Method of preserving leftover meat:**	
Freezing	99 (52.7)
Refrigeration	79 (42.0)
None	13 (6.9)
Drying	11 (5.9)
Smoking	10 (5.3)
Salting	1 (0.5)
**Food safety measures practised during work:**	
Disposing of spoilt meat	183 (97.3)
Avoiding work when suffering from diarrhoea	170 (90.4)
Avoiding work while having boils	167 (88.8)
Avoiding work when suffering from flu	161 (81.5)
Washing hands after using the bathroom	135 (71.8)
Avoiding keeping long nails	106 (56.4)
Wearing PPE	102 (54.3)
Washing hands before and after handling meat	83 (44.1)
**Abattoir workers hygiene practice**	
Good	161 (85.6)
Poor	27 (14.4)

As shown in Table 3, a greater proportion (57.0%) of abattoir workers who had good knowledge of abattoir hygiene practices were aged less than 30 years. Being a meat product retailer and having had a previous training on abattoir hygiene had a statistically significant relationship with the knowledge of good hygiene practice (p<0.05). It was shown that a greater proportion of workers (56.3%) who had good knowledge of hygiene practices were retailers.

**Table 3:** Relationship of socio-demographic and other variables with knowledge of good hygiene practice

Variable	Knowledge of good hygiene practice n=188 N (%)				

	Good (n=142)	Poor (n=46)	Total	χ^2^	p-value
**Age group (years)**					
≤30 years	82 (57.7)	23 (50.0)	105	0.846	0.358
>30years	60 (42.3)	23 (50.0)	83		
**Sex**					
Male	108 (76.1)	28 (60.9)	136	4.005	0.045
Female	34 (23.9)	18 (39.1)	52		
**Marital status**					
Married	70 (49.3)	27 (58.7)	97	1.229	0.268
Not married	72 (50.7)	19 (41.3)	91		
**Religion**					
Christianity	128 (90.1)	40 (87.0)	168	0.371	0.543
Islam	14 (9.9)	6 (13.0)	20		
**Level of education**					
<Secondary education	54 (38.0)	17 (37.0)	71	0.017	0.896
≥Secondary education	88 (62.0)	29 (63.0)	117		
**Work experience**					
≤5 years	76 (53.5)	26 (56.5)	102	0.126	0.723
>5 years	66 (46.5)	20 (43.5)	86		
**Category of worker**					
Retailer	80 (56.3)	13 (28.3)	93	10.957	**0.001** *
Butchers and others	62 (43.7)	33 (71.7)	95		
**Previous training on abattoir** **work**					
Yes	119 (83.8)	30 (65.2)	139	7.300	**0.007** *
No	23 (16.2)	16 (34.8)	49		

* Statistically significant P – value

Table 4 revealed the relationship between socio-demographic characteristics of the respondent and good hygiene practices. It shows that 130 (80.7%) abattoir workers who had good hygiene practices also had good knowledge about good hygiene practices compared to only 9.3% who had poor knowledge. Only knowledge had statistically significant association with good abattoir hygiene practices (p< 0.05). Good knowledge of good hygiene practices is the only statistically significant (AOR: 4.58; CI:1.8–11.7); p=0.001) predictor of good abattoir hygiene practice (Table 5). Those that have good knowledge have 5 times higher odds of engaging in good hygiene practices compared to those with poor knowledge.

**Table 4:** Relationship of socio-demographic and other variables with hygiene practices

Variable	Practice about good hygiene n=188 N (%)				

	Good (n=161)	Poor (n=27)	Total	χ^2^	p-value
**Age group (years)**					
≤30 years	92 (57.1)	13 (48.1)	105	0.759	0.384
>30years	69 (42.9)	14 (51.9)	83		
**Sex**					
Male	120 (74.5)	16 (59.3)	136	2.696	0.101
Female	41 (25.5)	11 (40.7)	52		
**Marital status**					
Married	81 (50.3)	16 (59.3)	97	0.741	0.389
Not married	80 (49.7)	11 (40.7)	91		
**Religion**					
Christianity	144 (89.4)	24 (88.9)	168	0.007	0.931
Islam	17 (10.6)	3 (11.1)	20		
**Level of education**					
<Secondary education	58 (36.0)	13 (48.1)	71	1.446	0.229
≥Secondary education	103 (64.0)	14 (51.9)	117		
**Work experience (years)**					
≤5 years	87 (54.0)	15 (55.6)	102	0.021	0.883
>5years	74 (46.0)	12 (44.4)	86		
**Category of worker**					
Retailer	81 (50.3)	12 (44.4)	93	0.318	0.573
Others	80 (49.7)	15 (55.6)	95		
**Previous training on abattoir work**					
No	30 (18.6)	9 (33.3)	139	3.039	0.081
Yes	131 (81.4)	18 (66.7)	49		
**Knowledge**					
Good	130 (80.7)	12 (44.4)	142	16.487	**<0.001** *
Poor	31 (19.3)	15 (55.6)	46		

* Statistically significant P - value

**Table 5:** Predictors of good hygiene practices

Independent Variables	AOR	p-value	95% C.I for AOR	
			
			Lower	Upper
**Sex**				
Male	1.29	0.65	0.43	3.86
Female	1			
**Previous training on abattoir work**				
No	0.60	0.37	0.19	1.83
Yes	1			
**Knowledge of good hygiene practices**				
Good	4.58	**0.001** *	1.80	11.66
Poor	1			

**Keys: C.I:** Confidence Interval; **AOR:** Adjusted Odds Ratio

* Statistical significance

## Discussion

This study was designed to investigate the knowledge and hygiene practices and its determinants among workers in the abattoir and slaughter slab in Abakaliki, Ebonyi State and to assess the facilities. Although there was overall good knowledge and hygienic practices, some essential hygiene practices were poorly practised in our study (Table 2 refers). A poor level of practice of good hygiene has been described by studies across six towns in Southeast Nigeria^20^, in North Central Nigeria^15^ and Kenya^31^. Interestingly, although 71.3% of our respondents knew that regular hand-washing was important (Table 1 refers), only 44.1% (Table 2) reportedly practised hand-washing before and after handling of meat. Since 71.8% (Table 2) reported washing hands after using the toilet, that may be what they consider adequate as regular hand-washing. The poor practice of hand-washing after handling of meat we found (Table 2 refers) is similar to studies in Oyo State, Nigeria^17^ where in 80% of the abattoir and slaughterhouses, there was poor practice and also in five North Central States in Nigeria^32^ where only 6% practised regular hand-washing. In contrast, the practice of hand-washing after operations was much higher (98.3% of the workers) in the abattoir in the Abuja area of the Federal Capital Territory (FCT), Nigeria.^33^. Among these, observation of the practice was employed only in the study in Oyo State. Careful and frequent hand-washing is advocated to reduce contamination8. Unwashed hands from poor personal hygiene may transmit microorganisms to wellcleaned surfaces before processing begins8 thus contaminating the meat while on the other hand, workers with poor hand-washing practices are at risk of getting infected with zoonoses^34^. A possible explanation for why less than half used soap and water to clean their work surfaces may be a knowledge gap in that area.

Overall, 75.5% had good knowledge of good hygiene practices and PPE in contrast to a study in Kwara state where 18% had good knowledge of food safety risks^35^. The percentage of workers in abattoir who wore aprons/overalls- (which was the commonest PPE used in our study)- in studies in Abuja FCT, Nigeria^33^ and Ethiopia9 was considerably higher (69.2% and 92.3% respectively) than the 54% of workers who wore PPE in our study. Lower percentages were reported by studies in Oyo State17 (32%), Kwara State35 (32.6%) and Kaduna State^36^ (18.2%) and five North Central States all in Nigeria^32^ (27.8%). Although the study in Abuja was self-reporting, their higher rates of PPE use and hand-washing after operations may be due to the reported attendance of public health education programs on abattoir operations by 54.2% of the workers. The overwhelmingly common practice of open dumping by the respondents appears to be a systemic problem of poor waste management practices in Nigeria^37^,^38^. Expectedly, the majority (81.3%) of the abattoir workers reported the apron as the most common PPE used, though this was much higher than was found in Southeast and North Central Nigeria^15^,^35^. In one of the studies in North central Nigeria^35^, safety boots was the most commonly used PPE. Protective clothing protects the meat from contamination and the workers from zoonoses.

The predictor of good hygiene practice being good knowledge accords with the observation by Alhaji and Baiwa^15^ which showed that workers who knew the correct definition of slaughterhouse hygiene were less likely to demonstrate poor preventive practices but differs from those of Junaidu^39^ where the predictor of good hygiene practice among the abattoir workers was a positive attitude not good knowledge. Our finding of previous training being significantly associated with good hygiene practice is similar to that of a study in Kenya where longer years of experience and increased capacity through training were significantly associated with good hygiene practices^40^. Training is expected to improve knowledge and practice when done adequately and other factors are in place. Only the Central meat market had available cold rooms and this may account for why the practice of storing meat in the cold room was not universal. The two cold rooms were not in the abattoir but adjoining streets and owned by individuals. The government has however built a cold room and other infrastructure in the Central meat market after this study soon to be commissioned, justifying the perception by a good majority of the respondents that the government is actively involved in ensuring good hygiene practice in the abattoir. The positive results of knowledge and hygiene practices may be attributed to the daily inspection of the slaughterhouses by officials of the Ministry of Health and Ministry of Environment as regulatory agencies and the occasional health talks given as 70.2% of them asserted government involvement in ensuring good hygiene practices. However, it is noted that observation of the workers is needed to confirm the reports of good hygiene practices. Good personal hygiene, other hygiene practices and standard facilities are all necessary for avoidance of contamination by microorganisms and transmission of zoonoses. The gaps we have identified in knowledge and practice, provide evidence for use in the design of intervention programmes for this group of workers.

## Conclusion

There is an overall good knowledge of hygiene practices as well as appreciably good hygiene practices possibly due to the health talks by the supervisory ministry officials. However, serious gaps in practice by more than half of the respondents were noted in some essential practices. The determinant of good hygienic practice was a good knowledge of hygienic practices. Notwithstanding the limitation from the self-reporting bias, this work contributes to our understanding of the knowledge and practice of hygienic practices with its determinants among workers in the abattoir and slaughter slab in Abakaliki and the practical implication suggests a basis for the immediate implementation of targeted interventions by government and stakeholders starting with training on the importance of good hygiene and sanitation. We recommend that a policy priority among policy-makers in the state be, developing and ensuring the implementation of policies that will safeguard our meat from contamination and protect the abattoir workers from zoonoses.

## Figures and Tables

**Figure 1:**
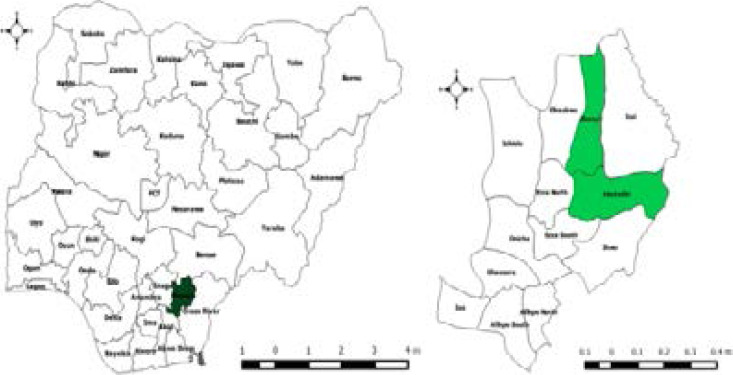
Maps of Nigeria showing Ebonyi State (left) and Ebonyi State (right) showing the LGAs where the study sites are located (in green)

**Table 6:** Availability of abattoir facilities

Facility	Central meat market	SlaughterslabSlaughter slab
Residential area	NA	NA
Local housing around abattoir	A	A
Lairage	NA	A
Slaughter hall	A	NA
Gut and tripe room	NA	NA
Detained meat room	NA	NA
Condemned meat room	A	NA
Offal room	NA	NA
Hide and skin room	NA	NA
Cutting room	NA	NA
Cold room	A	NA
Supply of hot and cold water under pressure	NA	NA
Veterinary inspection room	NA	NA
Disinfection facilities	NA	NA
Personnel welfare room	A	A
Veterinary office	A	NA
Cloak room	NA	NA
Facilities for condemned meat, offal or carcass disposal	NA	NA
Sufficient space for expansion	A	A
Freedom for flooding	A	A
Well	A	A
Tap	NA	NA
Borehole	A	A
Water closet	NA	NA
Pit latrine	NA	NA

Key: A=Available; NA=Not available
